# Publication of reviews synthesizing child health evidence (PORSCHE): a survey of authors to identify factors associated with publication in Cochrane and non-Cochrane sources

**DOI:** 10.1186/s13643-016-0276-7

**Published:** 2016-06-21

**Authors:** Lisa Hartling, Kassi Shave, Denise Thomson, Ricardo M. Fernandes, Aireen Wingert, Katrina Williams

**Affiliations:** Cochrane Child Health, University of Alberta, ECHA 4-472, 11405-87 Avenue, Edmonton, AB T6G 1C9 Canada; Alberta Research Centre for Health Evidence, University of Alberta, ECHA 4-472, 11405-87 Avenue, Edmonton, AB T6G 1C9 Canada; Cochrane Child Health, Cochrane Portugal, Department of Pediatrics and Clinical Pharmacology Unit, Lisbon Academic Medical Centre, Instituto de Medicina Molecular and University of Lisbon, Lisbon, Portugal; Cochrane Child Health, University of Melbourne, Royal Children’s Hospital and Murdoch Children’s Research Institute, Melbourne, Australia; Department of Pediatrics, Santa Maria Hospital, Lisbon Academic Medical Centre and Clinical Pharmacology Unit, Instituto de Medicina Molecular, Faculty of Medicine, University of Lisbon, Lisbon, Portugal; Department of Paediatrics, University of Melbourne, Developmental Medicine, Royal Children’s Hospital, Melbourne and Murdoch Children’s Research Institute, Melbourne, Australia

**Keywords:** Systematic reviews, Cochrane Collaboration, Publication, Survey

## Abstract

**Background:**

Cochrane Child Health maintains a register of child-relevant Cochrane systematic reviews (SRs) to provide a comprehensive source of high-quality evidence. However, a large number of SRs are published outside of The Cochrane Collaboration (Cochrane), impacting the comprehensiveness of the Cochrane Database of Systematic Reviews (CDSR). We surveyed authors who published child-relevant SRs with Cochrane and elsewhere in the medical literature to (1) understand their experiences in preparing and publishing SRs and (2) identify factors influencing choice of publication venue.

**Methods:**

We identified SRs published in the CDSR for the most recent complete year prior to our study (2013; *n* = 145). We searched the medical literature and randomly selected the same number of SRs published the same year. We developed an internet-based survey and contacted the corresponding author of each review via email. Data were analyzed descriptively. Qualitative analysis elicited common themes from open-ended questions.

**Results:**

Seventy-six (26 %) responded: 41 % Cochrane, 42 % non-Cochrane, and 17 % published in both venues. Among respondents who published their SR in both venues (*n* = 13), 46 % found it easier to publish in a non-Cochrane journal, 15 % easier with Cochrane, and 31 % similar. Main reasons for conducting SRs with Cochrane (*n* = 44) were Cochrane’s positive reputation (82 %) and good impact factor (66 %). Among respondents who published their SR in a non-Cochrane journal (*n* = 32), most frequent reasons for not conducting their SR with Cochrane were time required to follow Cochrane processes (25 %), lack of knowledge about how to conduct an SR with Cochrane (19 %), administrative processes (16 %), and perception that non-Cochrane journals yielded more interest (16 %). Among respondents who published their SR in a non-Cochrane journal (*n* = 32), 78 % did not register their review and 22 % did not prepare a protocol.

**Conclusions:**

Key reasons for publishing in Cochrane are its positive reputation and impact factor. Reasons for publishing in non-Cochrane sources include lack of familiarity or challenges with the Cochrane processes and desire to publish in a source more directly relevant to the topic of interest. End users looking for evidence in the form of SRs need to be aware that there is a vast number of SRs published across the medical literature. Efforts to optimize the identification of SRs in non-Cochrane sources (e.g., through effective labeling or protocol/review registration) and their content will help end users find the necessary synthesized evidence to support clinical practice.

**Electronic supplementary material:**

The online version of this article (doi:10.1186/s13643-016-0276-7) contains supplementary material, which is available to authorized users.

## Background

The Cochrane Collaboration (Cochrane) [http://www.cochrane.org/] was a pioneer in the area of evidence-based medicine. Established in 1993, it has been an advocate for the conduct and dissemination of systematic reviews of evidence on, initially, healthcare interventions and, more recently, in the area of diagnostic test accuracy. Cochrane’s systematic reviews are published in the online Cochrane Database of Systematic Reviews (CDSR), one of the databases included in The Cochrane Library [http://www.cochranelibrary.com/].

Individuals interested in conducting a systematic review through Cochrane are invited to register a title with one of 52 review groups which each focus on a specific area of health (e.g., acute respiratory infections, back and neck, breast cancer, etc.) [1]. Once the title is approved, review teams comprised of at least two authors prepare a protocol based on Cochrane guidance [2]. The protocol is submitted to the relevant review group which manages peer-review and publication of the protocol in the CDSR. After approval of the protocol, authors complete the systematic review; this is also submitted to the review group which manages peer-review and eventual publication in the CDSR. Additional details on the process for proposing and registering new reviews are available online [3]. The Cochrane Handbook provides detailed guidance for conducting systematic reviews; [2] further, Cochrane’s Central Editorial Unit [4] has established standards for protocols, reviews, and updates of reviews which are intended to guide the conduct and peer-review of Cochrane products [5].

Many systematic reviews are also conducted outside of Cochrane and published as technical reports by sponsoring agencies or in peer-reviewed medical journals. Many initiatives have emerged to increase the rigor of systematic reviews and transparency of reporting. One key initiative is the preferred reporting items for systematic reviews and meta-analyses (PRISMA) [6]. PRISMA provides a checklist to help improve reporting by systematic review authors and is endorsed by a number of editorial organizations and hundreds of journals [7].

In addition to review groups, Cochrane has networks pertaining to healthcare domains or populations of special interest [8]. These groups do not oversee the production of systematic reviews; rather, their functions are to facilitate linkages between Cochrane and external stakeholders, disseminate results of Cochrane reviews to the relevant stakeholders, and identify and tag titles, protocols, and reviews relevant to their scope [9]. They may also be involved in promoting the production of reviews, training, and conducting methodological research related to the production or dissemination of systematic reviews within their “field” or population of interest.

Cochrane Child Health is one such entity which was established to ensure that the unique health needs of children are reflected within The Cochrane Collaboration [http://www.cochrane.org/], [http://childhealth.cochrane.org/]. The Cochrane Library is a key output of Cochrane and an important source of synthesized evidence to inform healthcare delivery; however, to be most useful, it needs to include evidence that is up-to-date, comprehensive, of high methodological quality, and relevant to specific populations such as children. One crucial way of achieving this goal is to encourage authors of systematic reviews to publish their work in the CDSR. If authors find it easier and more appealing to publish their work in non-Cochrane arenas, this will dilute the comprehensiveness and therefore usefulness and applicability of the CDSR. Furthermore, it will be more difficult for clinicians and other decision-makers to quickly find relevant summarized data.

End users looking for evidence in the form of systematic reviews need to be aware that there are a vast number of reviews published outside of Cochrane. Previous research has shown that Cochrane reviews are of higher methodological quality; [10] it is also important to identify whether there are systematic differences in where authors choose to publish and the rationale for these choices. As the methods for systematic reviews of healthcare interventions have become more mainstream, and increasing numbers of clinicians and researchers have been trained in these methods and have experience completing systematic reviews, the facilitation provided by Cochrane may be less sought after and instead the processes that occur during review development may be perceived as a hindrance to timely publication.

Our goal was to understand the motivations of systematic reviewers for conducting and publishing their systematic reviews either with Cochrane (i.e., in the CDSR) or in other peer-reviewed journals. To meet our goal, we conducted a survey of authors who have completed and published child-relevant systematic reviews with the Cochrane Collaboration and elsewhere in the medical literature to (1) understand their experiences in preparing and publishing reviews and (2) identify factors that influenced choice of publication venue.

## Methods

We created a sample of recently published child-relevant systematic reviews. We identified all completed systematic reviews of healthcare interventions published in the CDSR for the most recent complete year prior to our study (2013; *n* = 145). We felt that capturing reviews published during a complete year would provide a broad representation to identify variables of interest. We then searched the medical literature (Additional file 1: Appendix A) and randomly selected the same number of child-relevant systematic reviews focusing on healthcare interventions published in the same year. We first identified 4855 records of systematic reviews from the same year. We defined a systematic review as all of the following (Additional file 1: Appendix B): (a) included a literature search that contained more than one named database, or one named database plus another sources (e.g., checking references, handsearching, contact with researchers to identify published studies, citation searching, internet searching, or other systematic attempts to identify potential studies); (b) reported inclusion and/or exclusion criteria; (c) described methods for study selection and data extraction; (d) assessed methodological quality of included studies; and (e) described or summarized results of the included/relevant studies. Non English-language reviews were excluded.

The list of systematic review records was randomly ordered, and we screened citations until we found an equivalent number of systematic reviews (*n* = 145) meeting our eligibility criteria, i.e., therapeutic intervention, child-relevant (Additional file 1: Appendix B). We defined a child-relevant therapeutic intervention as either of the following: (a) an intervention intended to improve the health and well-being of children; and (b) studies on breastfeeding or nutritional supplements for fetal and/or newborn health. Studies without pediatric outcomes were excluded, as well as primarily descriptive reports of studies in a given field with no synthesis of results (e.g., a descriptive analysis characterizing RCTs conducted in child health). We defined child-relevant as (a) intended to include children 0 to 18 years; (b) contained adults and children; or (c) studied an intervention/condition intended to improve health and well-being of children. Systematic reviews that only examined or reported maternal outcomes were excluded. We then identified the corresponding author of each review. We ensured that none of the authors surveyed were duplicates within and between lists (Cochrane vs. non-Cochrane). Since our non-Cochrane list was generated by screening records until we identified our target number, some of the Cochrane reviews may have also been published in a non-Cochrane source.

We developed an internet-based survey using Research Electronic Data Capture (REDCap) software [11] (Additional file 1: Appendix C). Study data were collected and managed using REDCap electronic data capture tools hosted at the University of Alberta [11]. REDCap is a secure, web-based application designed to support data capture for research studies, providing (1) an intuitive interface for validated data entry; (2) audit trails for tracking data manipulation and export procedures; (3) automated export procedures for seamless data downloads to common statistical packages; and (4) procedures for importing data from external sources [12].

Survey questions asked about: (1) why authors chose to publish their systematic reviews within or outside of the Cochrane Collaboration; (2) authors’ experiences preparing their systematic review; (3) whether they would undertake another review either within or outside of Cochrane; and (4) the reasoning and experiences of authors who chose to publish the same review in the CDSR and also in another journal. For most questions, we had pre-defined options that respondents could select as well as an “other” option with an opportunity for the respondent to elaborate with text descriptions. Prior to implementation, the survey was pilot tested among a convenience sample of five review authors (one was a co-author, RF, and four were independent of the study team) to ensure that the questions were worded appropriately and information was presented in a way that would elicit appropriate responses. The survey was revised based on their responses and feedback. Among the five individuals who pilot-tested the survey, three authors had published in both Cochrane and non-Cochrane sources, while two authors had published only in non-Cochrane sources.

We contacted the corresponding author of each review via email using the author’s published contact information. Participants were asked to respond to questions with respect to the specific publications (SR) we had identified. We identified 290 authors: 145 Cochrane authors and 145 non-Cochrane authors. We sent 290 emails with links to the survey directly to corresponding authors of the identified reviews. The survey was administered for 6 weeks, from April to May 2015. We sent two reminders following the initial email, each at 2-week intervals.

The study was approved by the University of Alberta’s research ethics board prior to implementation. All survey responses were anonymized. At the end of the survey, participants had the opportunity to enter their name in a draw for one of six US$50 Amazon gift cards (the names were entered on a separate web page and were not linked to the survey). We indicated that they would have about a 1 in 50 chance of winning based on our complete study sample; based on actual participation, the chance of winning was 1 in 13. Only one survey question was mandatory (i.e., respondents were required to complete the question before continuing with the survey); this was the first question asking authors to indicate if they had published with Cochrane, non-Cochrane or both. The remaining questions were based on response to this first question with slightly different questions asked of each group (Additional file 1: Appendix C). No other questions were mandatory in order to submit the survey. For incomplete questionnaires, we used the data for the questions that were answered; incomplete questions were marked as missing data. Respondents were able to amend answers at any time prior to submission of the survey (i.e., they could go backwards and forwards through the survey as they liked until they submitted). The survey invitation was linked to each participant’s email address. Participants were able to open the survey using the link as many times as they liked until they submitted the survey, at which time the link no longer worked. This ensured that we did not obtain repeat or duplicate responses.

Data were analyzed descriptively with univariate statistics using SPSS version 22.0. We divided respondents into three categories: (1) those who had only published their systematic review in the CDSR; (2) those who had published their review in both the CDSR and a non-Cochrane source (based on information provided by respondents); and (3) those who had only published their review in a non-Cochrane source. Qualitative analysis of the responses to open-ended survey questions was conducted to elicit common themes.

## Results

Seventy-six (26 %) individuals responded. Table 1 presents characteristics of the respondents; 41 % (*n* = 31) had published their review with Cochrane, 42 % (*n* = 32) in a non-Cochrane source, and 17 % (*n* = 13) in both. The majority of respondents were researchers (45 %; *n* = 34), clinician-scientists (32 %; *n* = 24), or clinicians (13 %; *n* = 10). A majority of participants had completed multiple systematic reviews (2–5, 41 % (*n* = 31); 6–10, 13 % (*n* = 10); 11–20, 5 % (*n* = 4); >20, 13 % (*n* = 10)), with 24 % (*n* = 18) of respondents having completed only one systematic review. A minority of the sample had no current involvement with Cochrane (38 %; *n* = 29); the proportion was higher for the authors who had only published their systematic review in a non-Cochrane source (75 %; *n* = 24).

**Table 1:** Characteristics of respondents and their intentions to participate in another systematic review

	All Authors *N* = 76 *n* (%)	Cochrane *N* = 31 *n* (%)	Both *N* = 13 *n* (%)	Non-Cochrane *N* = 32 *n* (%)
Primary professional role				
Clinician	10 (13.2)	4 (12.9)	3 (23.1)	3 (9.4)
Clinician-scientist	24 (31.6)	9 (29.0)	2 (15.4)	13 (40.6)
Researcher	34 (44.7)	14 (45.2)	8 (61.5)	12 (37.5)
Other	5 (6.6)	2 (6.5)	0 (0)	3 (9.4)
No response	3 (3.9)	2 (6.5)	0 (0)	1 (3.1)
Number of systematic reviews published as a lead or co-author				
1	18 (23.7)	7 (22.6)	2 (15.4)	9 (28.1)
2–5	31 (40.8)	12 (38.7)	4 (30.8)	15 (46.9)
6–10	10 (13.2)	5 (16.1)	3 (23.1)	2 (6.3)
11–20	4 (5.3)	2 (6.5)	1 (7.7)	1 (3.1)
>20	10 (13.2)	3 (9.7)	3 (23.1)	4 (12.5)
No response	3 (3.9)	2 (6.5)	0 (0)	1 (3.1)
Current involvement with The Cochrane Collaboration^a^				
Yes—author on current review (updating as required)	30 (39.5)	21 (67.7)	6 (46.2)	3 (9.4)
Yes—author on another review	33 (43.4)	21 (67.7)	8 (61.5)	4 (12.5)
Yes—employee of a Cochrane entity	6 (7.9)	5 (16.1)	0 (0)	1 (3.1)
Yes—other	7 (9.2)	4 (12.9)	2 (15.4)	1 (3.1)
No involvement	29 (38.2)	2 (6.5)	3 (23.1)	24 (75)
Would you participate as an author in another systematic review				
No	2 (2.6)	0 (0)	0 (0)	2 (6.3)
Yes—with Cochrane	55 (72.3)	28 (86.4)	10 (76.9)	17 (53.1)
Yes—outside Cochrane	15 (19.7)	1 (3.2)	3 (23.1)	11 (34.4)
No response	4 (5.3)	2 (6.5)	0 (0)	2 (6.3)
Reasons for not registering and publishing a future systematic review with The Cochrane Collaboration^a^				
Not interested due to administrative processes	8 (10.5)	0 (0)	1 (7.7)	7 (21.9)
Not interested due to reputation of Cochrane	0 (0)	0 (0)	0 (0)	0 (0)
Not interested due to time required to follow Cochrane’s processes	7 (9.2)	0 (0)	2 (15.4)	5 (15.6)
Not interested as peer-reviewed journal has higher impact factor	2 (2.6)	0 (0)	0 (0)	2 (6.3)
Source other than Cochrane yields more academic credit	0 (0)	0 (0)	0 (0)	0 (0)
Other	6 (7.9)	1 (3.2)	1 (7.7)	4 (12.5)

^a^More than one response permitted for this item

Among respondents who published their systematic review in both Cochrane and non-Cochrane journals (*n* = 13), the majority published in Cochrane first (77 %; *n* = 10). Nearly half reported it was easier to publish their systematic review in a non-Cochrane journal (46 %; *n* = 6); only 15 % (*n* = 2) found it easier with Cochrane, and 31 % (*n* = 4) rated ease of publishing equal between Cochrane and non-Cochrane journals. Respondents were divided in their rating of the timeliness of publishing with 31 % (*n* = 4) indicating it was more timely with Cochrane, 31 % (*n* = 4) more timely with a non-Cochrane journal, and 39 % (*n* = 5) indicating the same. A minority (31 %; *n* = 4) paid fees to publish their article in a peer-reviewed journal, and a similar proportion (31 %; *n* = 4) published in an open access journal.

**Fig. 1:**
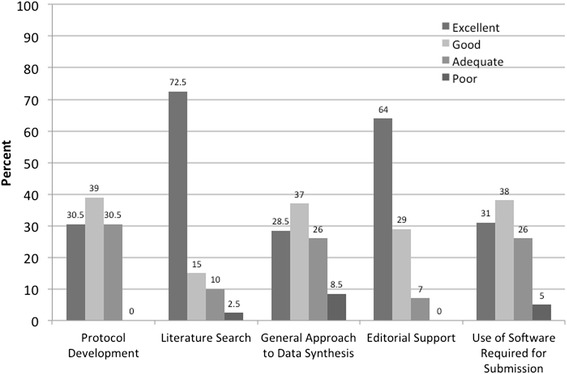
Respondents’ rating of support they received through the Cochrane Collaboration

Among respondents who published their systematic review in Cochrane (*n* = 44), the main reasons for conducting their systematic reviews with Cochrane were Cochrane’s positive reputation (82 %; *n* = 36) and good impact factor (66 %; *n* = 29) (respondents could select multiple response options). The majority who published their review with Cochrane were satisfied (48 %; *n* = 21) or very satisfied (32 %; *n* = 14). Respondents also rated the specific support they received (Fig. 1).

Table 2 summarizes responses of authors who published their systematic review in a non-Cochrane journal (*n* = 32). Nineteen percent (*n* = 6) had considered conducting their SR with Cochrane, while 75 % (*n* = 24) had not. The most frequent reasons for not conducting their systematic review with Cochrane were time required to follow Cochrane processes (25 %; *n* = 8), lack of knowledge about how to conduct a systematic review with Cochrane (19 %; *n* = 6), administrative processes (16 %; *n* = 5), and a perception that peer-reviewed journal publication yielded more interest (16 %; *n* = 5). Seventy-eight percent (*n* = 25) did not register their review and 22 % (*n* = 7) did not prepare a protocol before starting the systematic review. The most common reasons for not registering their systematic review were lack of knowledge about systematic review registers (31 %; *n* = 10), they did not think of it (19 %; *n* = 6), and not interested due to time required (13 %; *n* = 4). Respondents who published their systematic review in a non-Cochrane journal used the following Cochrane resources: Cochrane Library (50 %; *n* = 16), Cochrane Handbook for Systematic Reviewers of Interventions (50 %; *n* = 16), and a trials register (13 %; *n* = 4).

**Table 2:** Responses of authors who published in non-Cochrane journal (*n* = 32)

Considered registering title and conducting their SR with Cochrane	*N* (%)
Yes	6 (18.8)
No	24 (75)
No response	2 (6.3)
Reasons for not conducting SR with Cochrane^a^	
Did not know about Cochrane	1 (3.1)
Did not know how to conduct a systematic review with Cochrane	6 (18.8)
Administrative processes	5 (15.6)
Reputation of Cochrane	1 (3.1)
Time required to follow Cochrane processes	8 (25)
Peer-reviewed journal publication yields more interest	5 (15.6)
Wanted to reach a different audience	4 (12.5)
Procedures for publication more streamlined with peer-reviewed journal	4 (12.5)
Peer-reviewed journal has higher impact factor	1 (3.1)
Published work likely to be cited more outside of Cochrane	2 (6.3)
Source other than Cochrane yields more academic credit	1 (3.1)
Did not think of it	4 (12.5)
Other	4 (12.5)
Registered review with a SR register such as PROSPERO	
Yes	5 (15.6)
No	25 (78.1)
No response	2 (6.3)
Reasons for not registering review with a SR register^a^	
Did not know about SR registers	10 (31.3)
Did not know how to register a SR with a register	0 (0)
Not interested due to administrative processes	2 (6.3)
Not interested due to time required	4 (12.5)
Did not think of it	6 (18.8)
Other	5 (15.6)
Prepared protocol before starting SR	
Yes	24 (75)
No	7 (21.9)
No response	1 (3.1)
Published SR protocol in peer-reviewed journal	
Yes	4 (12.5)
No	20 (62.583.3)
No response	8 (25)
Paid publication fees to publish protocol in a peer-reviewed journal	
Yes	1 (3.1)
No	3 (9.4)
N/A	28 (87.5)
Reasons for not publishing SR protocol in a peer-reviewed journal^a^	
Did not know about publication of SR protocols	6 (18.8)
Did not know how to publish a SR protocol	0 (0)
Not interested due to administrative processes	2 (6.3)
Not interested due to time required	7 (21.9)
Did not see the value in publishing the protocol	4 (12.5)
Did not think of it	4 (12.5)
Other	3 (9.4)
Accessed specialized support of a librarian and/or information specialist	
Yes	21 (65.6)
No	10 (31.3)
No response	1 (3.1)
Accessed specialized support of a statistician	
Yes	13 (40.6)
No	18 (56.3)
No response	1 (3.1)
Specialized support of a librarian and/or information specialist would have been useful	
Yes	6 (18.8)
No	4 (12.5)
N/A	22 (68.8)
Specialized support of a statistician would have been useful	
Yes	2 (6.3)
No	16 (50)
N/A	14 (43.8)
Paid publication fees to publish SR in a peer-reviewed journal	
Yes	6 (18.8)
No	25 (78.1)
No response	1 (3.1)
Published SR in an open access journal	
Yes	9 (28.1)
No	22 (68.8)
No response	1 (3.1)
Aware of Cochrane SRs	
Yes	29 (90.6)
No	2 (6.3)
No response	1 (3.1)
Used Cochrane resources in preparing SR	
Yes	22 (68.8)
No	8 (25)
I did not know Cochrane had these resources	1 (3.1)
No response	1 (3.1)
Cochrane resources used in preparing SR	
The Cochrane Library	16 (50)
The Cochrane Handbook for Systematic Reviewers of Interventions	16 (50)
A Trials Register	4 (12.5)
Assistance from Cochrane staff	0 (0)
Other	4 (12.5)

*N/A* not applicable, *SR* systematic review

^a^More than one response permitted for this item

Overall, the majority (92 %; *n* = 70) of respondents indicated they would participate as an author in another systematic review. Overall, the majority (72 %; *n* = 52) indicated they would participate with Cochrane, with 20 % (*n* = 15) indicating outside of Cochrane. Among those not interested in participating with Cochrane (*n* = 15), the majority indicated that they were not interested due to the administrative processes (53 %; *n* = 8) or the time required to follow Cochrane processes (47 %; *n* = 7).

Themes that emerged from the open-ended responses, with supporting statements, are provided in Table 3. The following were some of the positive themes identified regarding The Cochrane Collaboration: Cochrane is recognized as producing high-quality, methodologically rigorous systematic reviews; Cochrane is considered an excellent organization with which to work; the support in producing systematic reviews offered through Cochrane is highly valued; and the resources offered through Cochrane are highly valued (e.g., RevMan, Handbook). The following were some criticisms of Cochrane processes for review production: application of systematic review standards varies by Cochrane Review Group; producing a Cochrane review is lengthy; some of the Cochrane requirements (e.g., search completed within 12 months) conflict with process (e.g., time for editorial review); time requirements often prohibit trainees (e.g., graduate students) from working with Cochrane; the increasing methodological requirements (e.g., GRADE) are adding complexity for authors, require additional training, and increase production time. Related to the last theme was an impression that the increasing methodological requirements may have an adverse impact on the readability and utility of the systematic reviews. Finally, the following themes help explain authors’ choice to seek publication outside of Cochrane: publication in non-Cochrane sources may reach a wider audience, or specific audience of interest (e.g., clinical specialty), and the scope of Cochrane is restrictive (e.g., only RCTs, clinical topics of interest, quantitative focus).

**Table 3:** Themes and supporting statements from open-ended questions

Theme	Sample supporting statements from survey respondents
Cochrane is recognized as producing high-quality, methodologically rigorous systematic reviews.	*“It can be a very long process, but the rigour of the reviews is ensured through much of this.”*
	*“I love the structured nature of the review and the way that all the questions you have a clearly answered within the handbook.”*
	*“Once completed, very happy. But the process was very long. However, this was for fine tuning and ensuring high quality, so justifiable in the end.”*
	*“The support and enthusiasm for high quality reviews is excellent. Best evidence reviews endorsed by Cochrane and reputable journals are fundamental to clinical decision-making.”*
	*“In the forefront of methodological development.”*
Cochrane is considered an excellent organization with which to work.	*“Fantastic. An excellent organisation to work with.”*
	*“All very professional and I always received responses in a timely manner.”*
	*“…my best colleagues and the people I respect more professionally are in the Cochrane Collaboration. I think that is much more than an editorial group, it is rather an approach to health care.”*
	*“I think the Cochrane collaboration is a great organisation. I think it is great that anybody can get involved in writing Cochrane reviews…”*
The support in producing systematic reviews offered through Cochrane is highly valued.	*“The access provided to an specialist to help develop the search and to run the searches is invaluable in the preparation of reviews.”*
	*“They were extremely patient, helpful, provide adequate and timely guidance for the statistical analysis and ensured the review's completion.”*
	*“They were very supportive throughout the process.”*
	*“The support given by the group was brilliant throughout.”*
	*“A very enjoyable and rewarding process. All the review writing software and other resources are easy to access and use.”*
	*“Far more editorial support than ordinary journals would give.”*
The process is standardized but application of standards varies by review group.	*“The process is standardised, though quality and quantity of the process is very different by each review group.”*
	*“I think that the process involved in getting these reviews done can be improved and streamlined across the review groups.”*
The process of producing a Cochrane systematic review is lengthy.	*“Good support. Total procedure was far too long.”*
	*“It took longer than I had initially anticipated.”*
	*“There is a long gap of time between finishing protocol and the first submission for review? This causes loss of momentum. This should be cut short and an intermediate stage of filling in the data should be introduced. A tutorial on analysis After data collection will be useful and decrease the dependence on the Cochrane expert author.”*
	*“Submitting a title and wait to hear if it is accepted. Submitting a protocol and the lengthy process that follow for it to be published. The lengthy peer review process of the review itself. I think that that submission from a third World country is not treated fairly.”*
	*“Takes more time than a traditional journal.”*
	*“The review process and clearing the protocol thought the specialty sub-groups is long and dampens momentum on projects. The ongoing commitment to review and update emerging evidence after the completion of the review is also daunting.”*
Some Cochrane requirements (e.g., search completed within 12 months) conflict with process (e.g., time for editorial review).	*“Peer review and iterations of the protocol and review added an unacceptable time lag to completing the review. The requirement for searches to be done within 6-12 months of publication of the review conflicts with the time involved in the editorial processes and peer review of the final review.”*
Time requirements often prohibit trainees (e.g., graduate students) from working with Cochrane.	*“Excellent support, but too long procedure to be included in regular PhD trajectories.”*
	*“Most of the SRs I have published over the years start as graduate student projects to understand what is available around the topic of their thesis. When we finally decide to pursue publication it just does not make sense to start a Cochrane process.”*
The increasing methodological requirements (e.g., GRADE) are adding complexity for authors, require additional training, and increase production time.	*“There should be a standard checklist for things to do especially with regards to use of GRADE criteria. Authors should be helped with this relatively new concept and be offered more help and encouragement.”*
	*“My only caveat is that it is hard at times to get every detail of the process exactly as the Cochrane editor wishes (though I understand the benefits as well as the disadvantages of a formulaic approach)”*
	*“Difficult as the rules and regulations are much more onerous I would probably advise people to now avoid the Cochrane process. MECIR guidelines are being interpreted by review groups in different ways and are putting the onus on authors to check about compliance to all the guidelines”*
	*“My experience is that people [are] in general scared by the amount of work needed.”*
The increasing methodological requirements may have an adverse impact on the readability and utility of the systematic reviews.	*“I think that the ever increasing requirements* e.g., *MECIR, summary of findings tables etc. is making the process for reviewers even more difficult and is having an adverse impact on the readability of the reviews.”*
Publication in non-Cochrane sources may reach a wider audience, or specific audience of interest (e.g., clinical specialty).	*“To increase coverage and readership”*
	*“To give wide audience to a topic we consider extremely important.”*
	*“It was an important clinical question and we used advanced methods which we thought would be of interest to journals”*
	*“We think CDSR is limited in some countries so publishing in paper journal may produce more impact to public and health care.”*
	*“More publications for the same work”*
The scope of Cochrane is restrictive (e.g., only randomized controlled trials, clinical topics of interest, quantitative focus).	*“Cochrane…very strict on what it accepts. They are also more [topics] which can fall outside the Cochrane remit”*
	*“We believe that SRs are justified even when no RCTs are available. Clinicians need to make decisions based on the best available evidence even if it not RCT driven. The Cochrane SRs do have as condition to include only RCTs.”*
	*“I think that The Cochrane Collaboration is often associated with very strict rules and regulations”*
	*“I dislike working with Cochrane because they are so slow and believe their way is the only/right way, even when there are alternative perspectives.”*

*CDSR* Cochrane Database of Systematic Reviews, *RCTs* randomized controlled trials, *SRs* systematic reviews

Suggestions offered by respondents to increase involvement and production of systematic reviews with Cochrane are summarized in Table 4. These include: streamlining processes; continuing to offer training and support; emphasizing the high-quality systematic reviews offered through Cochrane; increasing awareness about the high-impact factor of the CDSR; and addressing concerns of Cochrane being overly restrictive and quantitatively focused.

**Table 4:** Suggestions offered by respondents to increase involvement and production of systematic reviews with the Cochrane Collaboration

Theme	Sample supporting statements from survey respondents
Streamline processes.	*“…reducing the time involved in preparing and publishing with Cochrane”*
	*“Need to simplify the process and stop imposing new guidelines which add further complexity and make reviews less accessible to readers.”*
	*“Promoting rapid review methodology, exploring the use of crowd sourcing for supporting screening of citations, quicker turnaround time from submission of protocol/review manuscript to publication”*
Continue to offer training and support.	*“Continue to offer the workshops on a frequent basis.”*
	*“The training you can attend is invaluable and I have used these skills in a lot of places.”*
	*“We need more training and dissemination of the Cochrane work among clinicians and researchers.”*
	*“Get medical students involved.”*
Emphasize the high quality of systematic reviews offered through The Cochrane Collaboration.	*“It is a big challenge. I no longer view Cochrane as the gold standard. I believe Cochrane has set the standard but it is definitely possible to conduct a review which is as good as a Cochrane review and publish it much more rapidly outside of Cochrane. Personally I would always choose to do a Cochrane review from a loyalty perspective, the impact factor and the fact that I believe Cochrane peer referees and editors always consistently ensure a quality product.”*
Increase awareness about the high impact factor of the Cochrane Database of Systematic Reviews (CDSR).	*“Impact factor is not well-known.”*
	*“…change the impression that somehow a journal submission is ‘better’. My impression is that Cochrane reviews are often of higher quality on average than reviews published in journals. Yet, I would probably still choose a journal as a home for my reviews because journal publication often counts more toward tenure evaluation (or some other evaluation process). I’m not sure if Cochrane publication has the same weight.”*
Identify topics for review.	*“Review groups should develop priority lists for reviews if they already do not have such lists.”*
Address concerns of Cochrane being overly restrictive and quantitatively focused.	*“…greater emphasis on reviews other than effectiveness of interventions”*
	*“I think that Cochrane is seen as the gold standard in SRs which is wonderful for the collaboration. However, this may make it seem less attainable and less relatable to many groups and individuals. Attempting to still stress the high quality of reviews, while somewhat changing the very strict and quantitative based reputation can increase authorship and readership.”*

*SRs* systematic review

## Discussion

Our results provide some insights into authors’ experiences preparing and publishing systematic reviews, as well as factors that influence choice of publication arena, specifically Cochrane and non-Cochrane sources. Key reasons for publishing in Cochrane are its positive reputation and impact factor. Reasons for publishing in non-Cochrane sources include lack of familiarity or challenges with the Cochrane processes, and desire to publish in a source more directly relevant to the topic of interest. End users looking for evidence in the form of systematic reviews need to be aware that there are a vast number of systematic reviews published across the medical literature. The value of publishing systematic reviews in a single source (e.g., Cochrane) is that they are readily identifiable by end users. Efforts to optimize the identification of systematic reviews in non-Cochrane sources (e.g., through effective labeling or protocol/review registration) and their content will help end users find the necessary synthesized evidence to support clinical practice.

Cochrane was established over 20 years ago and its impact on the production of systematic reviews and development of systematic review methods is highly evident in the results of this survey. Even authors who did not conduct and publish their systematic reviews with Cochrane used Cochrane resources. The *Cochrane Handbook for Systematic Reviews of Interventions* is published and available online and widely used [2]. Our survey found that authors value the support offered through Cochrane in producing reviews, and the technical resources and guidance available.

Previous research comparing Cochrane and non-Cochrane reviews has found that Cochrane reviews are of superior methodological quality [10]. Cochrane processes (e.g., title registration, peer-review, and publication of protocols) were intended to increase transparency, reduce duplication, and optimize methodological rigor; however, there is little empiric evidence of the impact of the contributions of specific processes on the quality and uptake of Cochrane reviews. Moreover, the process of peer-review can be lengthy, resource intensive, and there is little evidence of its effectiveness in ensuring the quality of published research [13].

While considered more methodologically rigorous, it is unknown whether Cochrane reviews are perceived to be more accessible and usable, and whether there is a difference in terms of uptake and use of Cochrane vs. non-Cochrane reviews in clinical practice. Furthermore, respondents indicated that they published in non-Cochrane sources to reach a different target audience, in particular, members of their clinical specialty. Further research about publication venue for systematic reviews and where end users look for reviews may help ensure the Cochrane Library (and CDSR) is recognized as a “go-to” source for the most relevant and high-quality evidence.

Almost a quarter of non-Cochrane authors indicated that they did not prepare a protocol prior to conducting their review; this is consistent with earlier reports [10]. To our knowledge, there is no evidence that has linked lower methodological quality of systematic reviews without an a priori protocol (i.e., detailed methods beyond simply registering in a systematic reviews register such as PROSPERO). While some non-Cochrane respondents identified the administrative processes as a deterrent to publishing in the CDSR, there is currently a lack of evidence demonstrating that Cochrane reviews actually take longer to produce.

Our findings suggest a tension resulting from the motivation to produce high-quality systematic reviews and the administrative processes, perceived time, and methodological standards required to achieve this goal. Respondents provided some suggestions to address this challenge. Current investigations and interest in technologies and methods to increase efficiencies may help address some of the identified challenges. There is a growing interest as well as work being done within Cochrane to investigate technological approaches to streamlining review production, such as text mining and machine learning [14–17]. Covidence, a new software package for review production that links with RevMan and GRADEPro, has also been developed to manage all stages of review production and is revised regularly based on end user input [18]. The EMBASE project has investigated the use of crowdsourcing to increase efficiencies in screening records for CENTRAL [19–22]; similar approaches could be investigated within the production of reviews.

The timing of this study coincides with a widespread internal review of the structure and function of Cochrane’s component groups, including the review groups, methods groups, fields, and the consumer network. One of the key motivations for this internal review is to ensure that Cochrane continues to meet its mandate of producing high-quality, relevant, accessible evidence to inform healthcare decision-making. Our results may provide useful information as Cochrane continues to achieve its mandate [23].

This study is limited by the response rate which may affect generalizability; further, the invitation sent to participants had the name and title of one of the Directors of Cochrane Child Health which could have influenced response rate, who responded, and the nature of responses. A further concern is that some of the questions may not have been phrased in a balanced way which could have affected the responses we received, i.e., they may be perceived to be stated from a Cochrane perspective. For example, Cochrane authors were asked why they published with Cochrane, while non-Cochrane authors were not asked the equivalent question, i.e., they were asked why they did not publish in Cochrane, not why they published in a given journal. However, there was a similar number of respondents who had published in Cochrane and non-Cochrane sources, and thematic analyses indicated varied views with both positive and negative opinions. It is important to interpret the results in light of our desire to have a single “go-to” source for high-quality evidence in child health, or at a minimum that reviews of relevance are readily identifiable.

A further limitation is that the sample was derived from child-relevant systematic reviews published in a single year (2013) which may also limit generalizability. Opinions may vary for authors who were updating their reviews compared with authors who were conducting new reviews; however, we did not have adequate information to assess or comment on these differences. Finally, opinions and motives may vary by clinical topic (even within child health) and our selection of non-Cochrane reviews may not have matched the same topics covered in our sample of Cochrane reviews.

## Conclusions

We identified reasons that authors publish systematic reviews in Cochrane and non-Cochrane sources. These include the reputation of Cochrane, familiarity or challenges with Cochrane processes, and relevance of the publication venue to the topic of interest. The identification of Cochrane systematic reviews is easier because they reside in the same place (i.e., CDSR housed in The Cochrane Library). Efforts to optimize the identification of systematic reviews in non-Cochrane sources (e.g., through effective labeling or protocol/review registration) and their content will help end users find the necessary synthesized evidence to support clinical practice.

## Abbreviations

*CDSR* Cochrane Database of Systematic Reviews, *REDCap* research electronic data capture, *SRs* systematic reviews

## Additional files

Additional file 1: Appendix A: Search Strategy. Appendix B: Screening criteria for selection of non-Cochrane reviews. Appendix C: Survey Questions. (DOCX 51 kb)
