# Supplementary material for: Publication of reviews synthesizing child health evidence (PORSCHE): a survey of authors to identify factors associated with publication in Cochrane and non-Cochrane sources
Source: Syst Rev. 2016 Jun 21;5:104. doi: 10.1186/s13643-016-0276-7 (PMC4915186; doi:10.1186/s13643-016-0276-7)
Supplement: Additional file 1: — Appendix A: Search Strategy. Appendix B: Screening criteria for selection of non-Cochrane reviews. Appendix C: Survey Questions. (DOCX 51 kb) [file 13643_2016_276_MOESM1_ESM.docx]

**Appendix A: Search Strategy**

Database: Medline via Ovid <1946 to present>

| 1. exp Child/  2. exp Infant/  3. Adolescent/  4. exp Pediatrics/  5. (baby or babies or infant* or toddler* or child* or youth or teen* or adolescen* or p?ediatric*).tw |
| --- |
| 6. or/1-5 [MeSH and keywords for children] (3,019,594) |
| 7. medline.tw  8. systematic review.tw  9. meta analysis.pt |
| 10. or/7-9 [HIRU SR filter for max specificity] (97,003) |
| 11. and/6,10 [child terms + Spec SR filter] (17,510) |
| 12. Cochrane database of systematic reviews.jn [Journal name] (9,292)  13. 11 not 12 [removing Cochrane SRs from the search results] (15,105) |
| 14. exp animals/not humans.sh (3,869,588)  15. 13 not 14 [removing animal studies from the search results] (15,091) |
| 16. limit 15 to ed=“20130101-20140228” (1,996)  17. remove duplicates from 16 (1,959) |

Database: CINAHL via EBSCO

| S1. (MH "Child+") (391,051)  S2. (MH "Adolescence+") (290,069) |
| --- |
| S3. TX meta analysis  S4. PT review  S5. PT systematic review |
| S6. S3 OR S4 OR S5 (194,678) |
| S7. (S1 OR S2) AND S6 (28,382) |
| S8. S7 Limiters - Published Date: 20130101-20140231 (1,810) |
| S9. SO cochrane database of systematic reviews Limiters - Published Date: 20130101-20140231  (749)  S10. S7 NOT S9 Limiters - Published Date: 20130101-20140231 (1,694) |
| S11. S6 [Limiters - Published Date: 20130101-20140231; Age Groups: Infant, Newborn: birth-1 month, Infant: 1-23 months, Child, Preschool: 2-5 years, Child: 6-12 years, Adolescent: 13-18 years] (1,763)  S12. S11 NOT S9 (1,651) |
| S13. S10 OR S12 (1,694) |

Database: Web of Science

| 1. TI=(baby or babies or infant* or toddler* or child* or youth or teen* or adolescen* or p?ediatric*) (980,677) |
| --- |
| 2. TS=((meta analys*) OR meta-analys* OR metaanalys*) (141,517)  3. TS=(systematic review*) (71,440)  4. TS=medline (45,493) |
| 5. #4 OR #3 OR #2 (216,142) |
| 6. #5 AND #1 (10,744) |
| 7. SO=(Cochrane Database of Systematic Reviews) (6,676)  8. #6 NOT #7 (10,041) |
| 9. Timespan=2013-2014 (1,645) |

Database: PubMed via National Library of Medicine

| (((((((((("Infant"[Mesh]) OR "Child"[Mesh]) OR "Adolescent"[Mesh]) OR "Pediatrics"[Mesh])) OR ((baby or babies or infant or toddler or child or youth or teen or adolescent or pediatric[Title/Abstract])))) AND (((Medline[Title/Abstract]) OR ((systematic[Title/Abstract]) AND review[Title/Abstract])) OR meta analysis[Publication Type])) AND ( "2013/09/01"[PDat] : "2014/02/28"[PDat] ))) NOT "Cochrane Database Syst Rev"[Journal] |
| --- |

**Appendix B: Screening criteria for selection of non-Cochrane reviews**

| **CRITERIA** | **SUB-CRITERIA** | **DECISION**  **Yes - Include;**  **No - Exclude;**  **Unclear - Include** |  |
| --- | --- | --- | --- |
| **A) Publication is a Systematic Review - based on criteria from DARE (**<http://www.crd.york.ac.uk/CRDWeb/AboutPage.asp>**) and the Cochrane Handbook^1^** | | | |
| **If you answer "No" for any of the SR criteria below, the review can be excluded without further screening.** | | | |
| Search | 1. Was the search adequate?   **YES, if 1 or 2:**  1) >1 named database, OR  2) 1 named database **plus** any of the following:   - Checking references - Handsearching - Contact with researchers to identify unpublished studies - Citation searching - Internet searching - Other systematic attempts to identify potential studies   If number of databases searched is given, but individual databases are not named, select 'Yes'.  **NO (either or both of the following):**   - No search is reported - Only 1 named database was searched |  |  |
| Criteria | 1. Were inclusion and/or exclusion criteria reported?   **Unclear:**  If the authors indicate that they used pre-specified inclusion/exclusion criteria, but do not report them. |  |  |
| Methods | 1. Were methods described for study selection and data extraction? |  |  |
| Quality assessment | 1. Was the quality of the included studies assessed?   **YES (any of the following):**   - formal quality assessment using a tool or components common to systematic reviews (e.g., Risk of Bias tool, Newcastle-Ottawa Scale, allocation concealment, blinding) - some other form of critical appraisal (i.e., consideration of study design and methods) - discussion of study design limitations of the individual studies, or hierarchy of evidence |  |  |
| Synthesis | 1. Was there a description or summary of results of the included/relevant studies?   **YES (any of the following):**   - A statistical synthesis - A narrative synthesis - A description of individual or overall study results either in text or tables (including, each study is described separately with no attempt to combine the results in a statistical or narrative synthesis) - If a review fails to identify any studies, or only 1 study   **NO:**   - A summary of the included studies is presented only as a conclusion in the abstract, or within the discussion, but not as a stand-alone section |  |  |
| **B) Publication is relevant to child health - based on Bow 2010 paper^2^** | | | |
| Children | 1. Does the review include children?   **YES (any the following):**   - Intended to include children 0-18y - If the review does not specify age, but the condition is relevant to children - If the review contains adult and children (mixed population) |  |  |
| Interventions/ Conditions | 1. Does the review examine interventions/conditions relevant to children?   **YES (any of the following):**   - Studied an intervention intended to improve health and well-being of children (e.g. smoking programs for family and caregivers, family-centred care for hospitalized children, parenting programs for psychosocial outcomes in adolescents) - Studies on breastfeeding or nutritional supplements for baby during pregnancy   Pediatric outcomes must be reported (e.g. a review does not qualify for inclusion if it only examined or reported maternal outcomes)  **NO:**   - The review focuses on pregnancy |  |  |

**Note:** Exclude if the report is a descriptive analysis of studies in a given field. For example, a descriptive analysis characterizing RCTs conducted in child health. These studies may conduct a "comprehensive" search and have pre-specific inclusion/exclusion criteria and methods; however, their intent is to characterize the research in an area (e.g., quality, quantity, scope) and not to synthesize results from the studies.

^1^ Higgins JPT, Green S (editors). Cochrane Handbook for Systematic Reviews of Interventions Version 5.1.0 [updated March 2011]. The Cochrane Collaboration, 2011. Available from www.cochrane-handbook.org

^2^ Bow S, Klassen J, Chisholm A, et al. A descriptive analysis of child-relevant systematic reviews in the cochrane database of systematic reviews. BMC pediatrics 2010;10(34. PMID:20487565. 2881081: 2881081.

**Appendix C: Survey Questions**

The survey was developed in and administered through REDCap. There were three streams of questions depending on how authors responded to Question 1 below. When respondents selected “other” when it was available as a response option, a new field opened asking for more details. Response options for closed-ended questions are shown below. Where there are no response options, the question was open-ended, and respondents could enter response in a free text field.

**QUESTIONS ASKED OF ALL AUTHORS**

**1. Where have you published your systematic review?**

(A) Only with The Cochrane Collaboration

(B) Both Cochrane and non-Cochrane peer-reviewed journals

(C) Only in a non-Cochrane peer-reviewed journal

**2. Did this publication represent:**

(A) The first time this systematic review was published

(B) An update of an earlier version

**QUESTIONS ASKED OF COCHRANE AUTHORS ONLY (1A) AND AUTHORS WHO HAD PUBLISHED IN BOTH COCHRANE AND NON-COCHRANE PEER-REVIEWED JOURNALS (1B)**

**Why did you choose to conduct the above named systematic review with The Cochrane Collaboration?**

□ The Cochrane Collaboration has a positive reputation

□ The Cochrane Collaboration offered support that helped me produce the review

□ The Cochrane Database of Systematic Reviews has a good impact factor

□ Co-authors on the review wanted to publish with Cochrane

□ Other

**Please elaborate on other reasons you chose to conduct your systematic review with The Cochrane Collaboration**

**Please rate the support you received from the Cochrane Review Group for the following:**

***Protocol Development:*** N/A, Poor, Adequate, Good, Excellent

***Literature Search:*** N/A, Poor, Adequate, Good, Excellent

***General Approach to Data Synthesis:*** N/A, Poor, Adequate, Good, Excellent

***Statistical Analysis of the Data:*** N/A, Poor, Adequate, Good, Excellent

***Editorial Support:*** N/A, Poor, Adequate, Good, Excellent

**Do you have any comments on the support you received from your Cochrane Review Group?**

**How long did it take to prepare your protocol (not including time for the editorial and peer-review process)?** Please specify unit of time, e.g., 1 year, 3.5 months, 20 days, etc.

**How long did it take for your protocol to be published from the time you first submitted it (including time for the editorial and peer-review process)?** Please specify unit of time, e.g., 1 year, 3.5 months, 20 days, etc.

**How long did to take to complete the review (not including time for the editorial and peer-review process and not including time to prepare the protocol)?** Please specify unit of time, e.g., 1 year, 3.5 months, 20 days, etc.

**How satisfied were you with the overall process of preparing a review with Cochrane**

Unsatisfied, somewhat satisfied, satisfied, very satisfied

**Do you have any comments regarding the process of preparing your review with Cochrane?**

**QUESTIONS ASKED OF AUTHORS WHO PUBLISHED IN BOTH COCHRANE AND NON-COCHRANE PEER-REVIEWED JOURNALS (1B)**

**Why did you choose to publish your systematic review with both a Cochrane and a non-Cochrane peer-reviewed journal?**

**Where did you publish your systematic review first?**

(A) Cochrane

(B) Peer-reviewed journal

**What factors determined where you first published your review?**

**How would you rate the ease of publishing your systematic review?**

(A) Easier with Cochrane

(B) Easier with the peer-reviewed journal

(C) The same

**How would you rate the timeliness of publishing your systematic review?**

(A) More timely with Cochrane

(B) More timely with the peer-reviewed journal

(C) The same

**Did you pay publication fees to publish your article in the peer-reviewed journal?**

(A) Yes

(B) No

**Did you publish your systematic review in an open access journal?**

(A) Yes

(B) No

**Which open access journal did you publish your systematic review in?**

**How long did it take for your review to be published in a non-Cochrane peer-reviewed journal from the time you first submitted it (including time for editorial and peer-review process and submissions to multiple journals if applicable)?** Please specify unit of time, e.g., 1 year, 3.5 months, 20 days, etc.

**How many peer-reviewed journals did you submit your review to before it was published?**

**QUESTIONS ASKED OF AUTHORS WHO HAD PUBLISHED ONLY IN A NON-COCHRANE PEER-REVIEWED JOURNAL (1C)**

**Did you consider registering your title and conducting your systematic review with The Cochrane Collaboration?**

(A) Yes

(B) No

**Why did you not choose to conduct your systematic review with The Cochrane Collaboration (check all that apply)?**

□ Didn’t know about The Cochrane Collaboration

□ Didn’t know how to conduct a systematic review with The Cochrane Collaboration

□ Administrative processes

□ Reputation of The Cochrane Collaboration

□ Time required to follow The Cochrane Collaboration’s processes

□ Peer-reviewed journal publication yields more interest

□ I wanted to reach a different audience

□ Procedures for publication are more streamlined with a peer-reviewed journal

□ Peer-reviewed journal has higher impact factor

□ Published work likely to be cited more outside of Cochrane

□ Source other than Cochrane yields more academic credit

□ Didn’t think of it

□ Other

**Please elaborate on other reasons that lead you to not conduct your systematic review with The Cochrane Collaboration?**

**Did you register your review with a systematic reviews register such as PROSPERO?**

(A) Yes

(B) No

**Why did you choose to not register your review with a systematic reviews register?**

□ Didn’t know about systematic reviews registers

□ Didn’t know how to register a systematic review with a register

□ Not interested due to administrative processes

□ Not interested due to time required

□ Didn’t think of it

□ Other

**What other reasons lead you to choose to not register your review with a systematic reviews register?**

**Did you prepare a protocol before starting your systematic review?**

(A) Yes

(B) No

**How long did it take to prepare your protocol (not including time for the editorial and peer-review process if it was published)?** Please specify unit of time, e.g., 1 year, 3.5 months, 20 days, etc.

**Did you publish your systematic review protocol in a peer-reviewed journal?**

(A) Yes

(B) No

**How long did it take for your protocol to be published from the time you first submitted it (including time for the editorial and peer-review process and submissions to multiple journals if applicable)?** Please specify unit of time, e.g., 1 year, 3.5 months, 20 days, etc.

**Please specify the peer-reviewed journal where you published your systematic review protocol.**

**Did you pay publication fees to publish your protocol in a peer-reviewed journal?**

(A) Yes

(B) No

**Why did you choose to not publish your systematic review protocol in a peer-reviewed journal?**

□ Didn’t know about publication of systematic review protocols

□ Didn’t know how to publish a systematic review protocol

□ Not interested due to administrative processes

□ Not interested due to time required

□ Didn’t see the value in publishing the protocol

□ Didn’t think of it

□ Other

**What other reasons lead you to not publish your systematic review protocol in a peer-reviewed journal?**

**Did you have any of the below specialized resources to support your review?**

***Librarian and/or Information Specialist:*** Yes, No

***Statistician:*** Yes, No

**Would a librarian/information specialist have been useful to you?**

(A) Yes

(B) No

**Would a statistician have been useful to you?**

(A) Yes

(B) No

**How long did it take to complete the review (not including time for the editorial and peer-review process and not including time to prepare the protocol if applicable)?** Please specify unit of time, e.g., 1 year, 3.5 months, 20 days, etc.

**How long did it take for your review to be published from the time you first submitted it (including time for the editorial and peer-review process and submission to multiple journals if applicable)?** Please specify unit of time, e.g., 1 year, 3.5 months, 20 days, etc.

**Did you pay publication fees to publish your article in a peer-reviewed journal?**

(A) Yes

(B) No

**Did you publish your systematic review in an open access journal?**

(A) Yes

(B) No

**In which open access journal did you publish your systematic review?**

**How many peer-reviewed journals did you submit your review to before it was published?**

**Are you aware of The Cochrane Collaboration’s systematic reviews?**

(A) Yes

(B) No

**Did you use any of The Cochrane Collaboration’s resources, such as the Cochrane Library, the Cochrane Handbook, a Trials Register from a particular review group, assistance from Cochrane staff, etc., in preparing your systematic review?**

(A) Yes

(B) No

(C) I did not know Cochrane had these resources

**Which of the following Cochrane resources did you use (check all that apply)?**

□ The Cochrane Library

□ The Cochrane Handbook for Systematic Reviewers of Interventions

□ A Trials Register

□ Assistance from Cochrane Staff

□ Other

**Please elaborate on the other Cochrane resources you used.**

**QUESTIONS ASKED OF ALL AUTHORS**

**Would you participate as an author in another systematic review?**

(A) Yes – with Cochrane

(B) Yes – with Cochrane but not with the same Review Group

(C) Yes – outside Cochrane

(D) No

**Why would you not participate as an author in another systematic review?**

**Why may you not consider registering and publishing a future systematic review with The Cochrane Collaboration?**

□ Not interested due to administrative processes

□ Not interested due to reputation of The Cochrane Collaboration

□ Not interested due to time required to follow The Cochrane Collaboration’s processes

□ Not interested as peer-reviewed journal has higher impact factor

□ Source other than Cochrane yields more academic credit

□ Other

**Please elaborate on why you are not interested due to the reputation of The Cochrane Collaboration.**

**Please elaborate on other reasons you would not consider registering and publishing a future systematic review with The Cochrane Collaboration.**

**Please describe your primary professional role.**

(A) Clinician

(B) Clinician-Scientist

(C) Statistician

(D) Researcher

(E) Information Specialist

(F) Other

**Please describe your primary professional role.**

**How many systematic reviews have you published as a lead or co-author?**

(A) 1

(B) 2-5

(C) 6-10

(D) 11-20

(E) >20

**Do you have current involvement with The Cochrane Collaboration?** (Check all applicable).

□ Yes – author on current review (updating as required)

□ Yes – author on another review

□ Yes – employee of a Cochrane entity

□ Yes – other

□ No involvement

**Please specify your other current involvement with The Cochrane Collaboration.**

**Do you have suggestions for how to encourage authors to publish their reviews with The Cochrane Collaboration?**

**Do you have any additional comments?**
